# 1,25(OH)_2_D_3_ Alleviates Aβ(25-35)-Induced Tau Hyperphosphorylation, Excessive Reactive Oxygen Species, and Apoptosis Through Interplay with Glial Cell Line-Derived Neurotrophic Factor Signaling in SH-SY5Y Cells

**DOI:** 10.3390/ijms21124215

**Published:** 2020-06-13

**Authors:** Ching-I Lin, Yi-Chen Chang, Ning-Jo Kao, Wei-Ju Lee, Tzu-Wen Cross, Shyh-Hsiang Lin

**Affiliations:** 1 Department of Nutrition and Health Sciences, Kainan University, Taoyuan 338, Taiwan; cilin@mail.knu.edu.tw (C.-IL.); mimikao0401@gmail.com (N.-J.K.); 2 School of Nutrition and Health Sciences, Taipei Medical University, Taipei 11042, Taiwan; m507101011@tmu.edu.tw; 3 School of Food Safety, Taipei Medical University, Taipei 11042, Taiwan; weijulee@tmu.edu.tw; 4 Department of Nutrition Science, Purdue University, West Lafayette, IN 47907, USA; tlcross@purdue.edu; 5 Master Program in Food Safety, Taipei Medical University, Taipei 11042, Taiwan

**Keywords:** Amyloid beta, Alzheimer’s disease, vitamin D, vitamin D receptor, GDNF, tau protein

## Abstract

Amyloid beta (Aβ) accumulation in the brain is one of the major pathological features of Alzheimer’s disease. The active form of vitamin D (1,25(OH)_2_D_3_), which acts via its nuclear hormone receptor, vitamin D receptor (VDR), has been implicated in the treatment of Aβ pathology, and is thus considered as a neuroprotective agent. However, its underlying molecular mechanisms of action are not yet fully understood. Here, we aim to investigate whether the molecular mechanisms of 1,25(OH)_2_D_3_ in ameliorating Aβ toxicity involve an interplay of glial cell line-derived neurotrophic factor (GDNF)-signaling in SH-SY5Y cells. Cells were treated with Aβ(25-35) as the source of toxicity, followed by the addition of 1,25(OH)_2_D_3_ with or without the GDNF inhibitor, heparinase III. The results show that 1,25(OH)_2_D_3_ modulated Aβ-induced reactive oxygen species, apoptosis, and tau protein hyperphosphorylation in SH-SY5Y cells. Additionally, 1,25(OH)_2_D_3_ restored the decreasing GDNF and the inhibited phosphorylation of the phosphatidylinositol 3 kinase (PI3K)/protein kinase B (Akt)/glycogen synthase kinase-3β (GSK-3β) protein expressions. In the presence of heparinase III, these damaging effects evoked by Aβ were not abolished by 1,25(OH)_2_D_3._ It appears 1,25(OH)_2_D_3_ is beneficial for the alleviation of Aβ neurotoxicity, and it might elicit its neuroprotection against Aβ neurotoxicity through an interplay with GDNF-signaling.

## 1. Introduction

Alzheimer’s disease (AD) is one of the most commonly occurring neurodegenerative diseases, and it is characterized by two pathologic feature: aberrant deposition of amyloid beta (Aβ) in extracellular plaques and intracellular accumulation of phosphorylated tau proteins in the brain [1,2,3]. This disease clinically presents slow progressive memory loss and cognitive deficits. Whether aberrant Aβ and tau proteins are key mechanisms in response to the AD-associated neuronal loss and death is still poorly understood. Aberrant Aβ has been gaining increasing attention due to the possibility of AD pathogenesis being initiated by this event and as a probable mediator of tau-pathology [4], although there is controversy concerning whether aberrant Aβ is a prerequisite for the hyperphosphorylation of tau protein [5]. The neurotoxicity of Aβ(1-42), which is the predominant Aβ species, has been addressed in both in vivo and in vitro models, and putative underlying mechanisms of its actions include reactive oxygen species (ROS) production and cell apoptosis [6,7]. A direct link between Aβ neurotoxicity and ROS production has been increasingly shown with methionine located at residue 35 (methionine 35) of Aβ(1-42) [8]. It has been proposed that methionine 35 of Aβ has the greatest vulnerability to oxidation, and is prone to being attacked by various radicals [7,8]. In in vitro and in vivo experiments, when methionine 35 residue of Aβ(25-35) and Aβ (1-42) was substituted with norleucine, oxidative stress and neurotoxicity were prevented [7,9]. It is noted that attention has been specifically directed to neurotoxicity and apoptotic cell death induced by Aβ(25-35), the short fragment of full length Aβ (1-42) with a retained methionine 35 residue and greater toxicity [10]. In this context, methionine 35 residue of Aβ(25-35) has been proposed to be an important contributor to apoptotic cell death involved in Aβ(25-35)-mediated neurotoxic properties [11]. Collectively, Aβ itself, under pathological conditions, has high potential to promote ROS production, which in turn may exacerbate the damage of oxidative stress (including lipid peroxidation). Notably, enhanced oxidative stress has been implicated in the pathogenesis of AD [12]. It is probable that excessive production of ROS in response to aberrant Aβ also activates apoptosis, suggesting an interplay among the production of ROS, the induction of apoptosis, and the toxicity of Aβ at the cellular level. A putative cellular signaling pathway that regulates this interplay is assumed to be the PI3K/Akt/GSK-3β pathway [13,14,15]. Thus, it is possible that antioxidants capable of mediating this pathway may be potential therapeutic targets for modulating Aβ-induced neurotoxicity. 

Intriguingly, vitamin D, particularly its active form, 1α,25-dihydroxyvitamin D3 (1,25(OH)_2_D_3_), has emerged recently as a new, attractive agent for combating AD [16,17]. Epidemiological studies strongly associated higher vitamin D intake with a lower risk of AD [18]. A significant decrease in the vitamin D repository in the body was found in patients with mild cognitive impairment status [19]. In addition, vitamin D deficiency occurring in older adults is closely linked to neurological dysfunction and cognitive decline [20]. Collectively, it is apparent that the active form of vitamin D (1,25(OH)_2_D_3_) may be of therapeutic value in AD. It is well-documented that 1,25(OH)_2_D_3_ exerts its actions via its nuclear hormone receptor, the vitamin D receptor (VDR), known as a transcription factor [21]. The interaction of VDR with its ligand 1,25(OH)_2_D_3_ is responsible for regulation of the target gene transcription in relation to a wide array of physiological functions, such as calcium homeostasis, cell proliferation, and cell differentiation [21,22]. VDRs are widely distributed in the brain, suggesting the importance of vitamin D in maintaining neurophysiological functions of the brain including neurodevelopment, neuronal proliferation, and neuronal survival [16,22]. The neuroprotective potential of 1,25(OH)_2_D_3_ against AD may be mediated by multiple mechanisms, such as anti-inflammatory effects [23], antioxidant properties [24], and enhancement of Aβ clearance [25]. However, the molecular mechanisms by which1,25(OH)_2_D_3_ exerts its neuroprotective effects have not yet been completely elucidated. One possible mode of action may be related to the regulation of the neurotrophic factors such as the brain glial cell line-derived neurotrophic factor (GDNF) at the cellular level [22].

Evidence exists that the 1,25(OH)_2_D_3_-VDR pathway plays a key role in the stimulation of the synthesis of GDNF, which promotes neuronal functions including neuron survival and differentiation, and neurite branching in the brain [26,27,28,29,30]. Interestingly, it was shown that a deficiency of vitamin D in the brain causes a decrease in GDNF expression [31]. Likewise, decreased GDNF levels in the blood and increased GDNF levels in cerebrospinal fluid samples of AD patients have been detected, suggesting that GDNF could be a putative target protein for AD-related pathology [32]. Therefore, the common protein, GDNF, involving two distinct cellular events, the 1,25(OH)_2_D_3_-VDR pathway and AD-pathological targets, is conducive for positing a mechanistic hypothesis that the protective mechanisms of 1,25(OH)_2_D_3_ against Aβ neurotoxicity could be driven by VDR-ligand 1,25(OH)_2_D_3_/GDNF interplay that targets the PI3K/AKT/GSK-3β pathway. To test this hypothesis, the human neuroblastoma cell line, SH-SY5Y, was pre-incubated with Aβ(25-35) followed by treatment with the addition of 1,25(OH)_2_D_3_. In the present study, cell viability, intracellular ROS, apoptosis, and phosphorylated tau protein were determined to reflect the putative neuroprotective effects of 1,25(OH)_2_D_3_ on modulating Aβ-related pathology. Moreover, Western blot analysis was employed to examine the Aβ-induced alterations in cellular mediators, including VDR and GDNF, and molecules of the PI3K/AKT/GSK-3β signaling pathway in SH-SY5Y cells.

## 2. Results

### 2.1. Effects of 1,25(OH)_2_D_3_ on Cell Morphology, Cell Viability, and Protein Expression of VDR and GDNF After Aβ(25-35) Treatment

**Figure 1:**
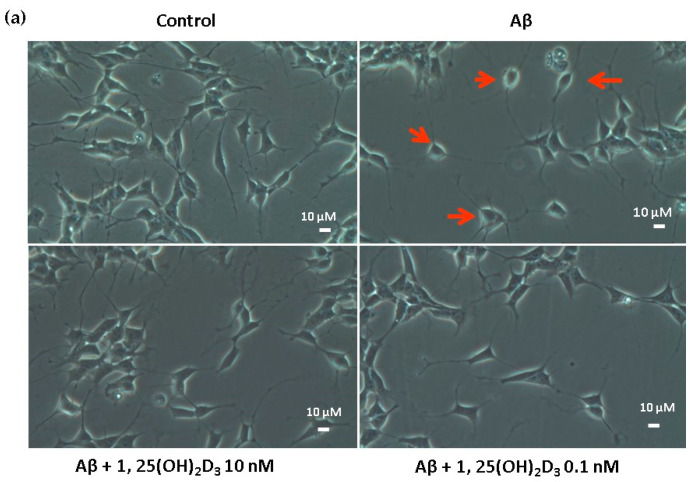

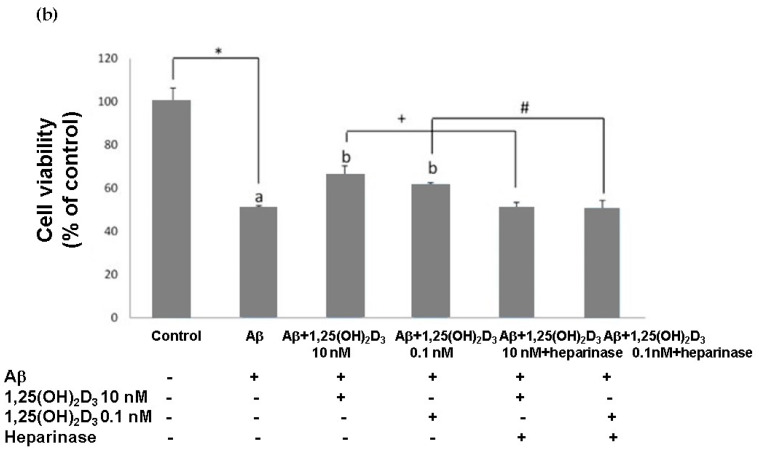
Effects of 1,25(OH)_2_D_3_ on Aβ-induced changes in SH-SY5Y cell morphology and cell viability. (**a**) SH-SY5Y cell morphology. Bar, 10 μM. Images were analyzed with SPOT 4.7 Advanced software. The arrows indicate the shorter neurite outgrowth of SH-SY5Y cells after the Aβ(25-35) challenge. (**b**) SH-SY5Y cell viability was analyzed by an MTT assay. SH-SY5Y cells were incubated with 1 μM Aβ(25-35) prior to the addition of 0.1 and 10 nM 1,25(OH)_2_D_3_ with or without heparinase III for 24 h. Data are presented as the mean ± SD of three experiments, and each experiment included triplicate repeats. *^,+,#^ Significantly differs between the two groups (statistical analysis was performed using Student’s t test). Bars of Aβ, Aβ + 0.1 nM 1,25(OH)_2_D_3_, and Aβ + 10 nM 1,25(OH)_2_D_3_ with different letters significantly differ (*p* < 0.05) (statistical analysis was performed using one-way analysis of variance (ANOVA) with Duncan’s post-hoc analysis).

**Figure 2:**
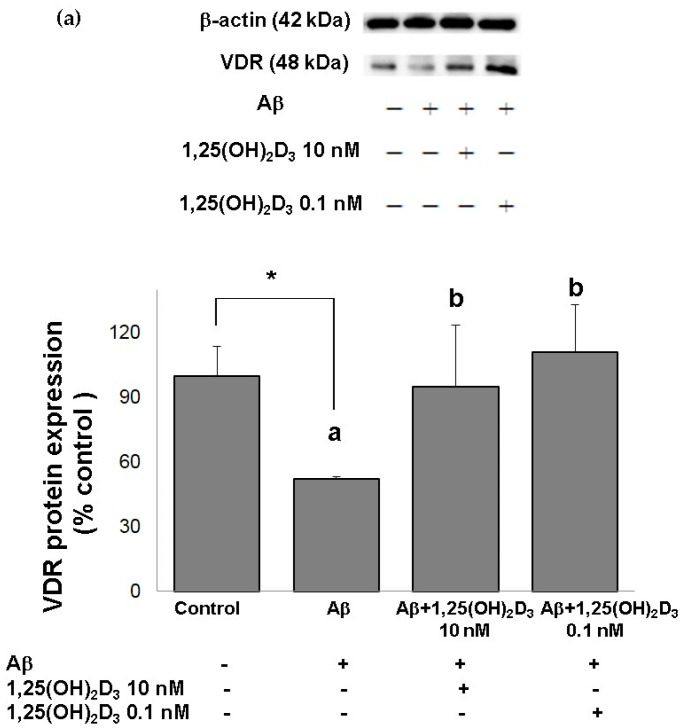

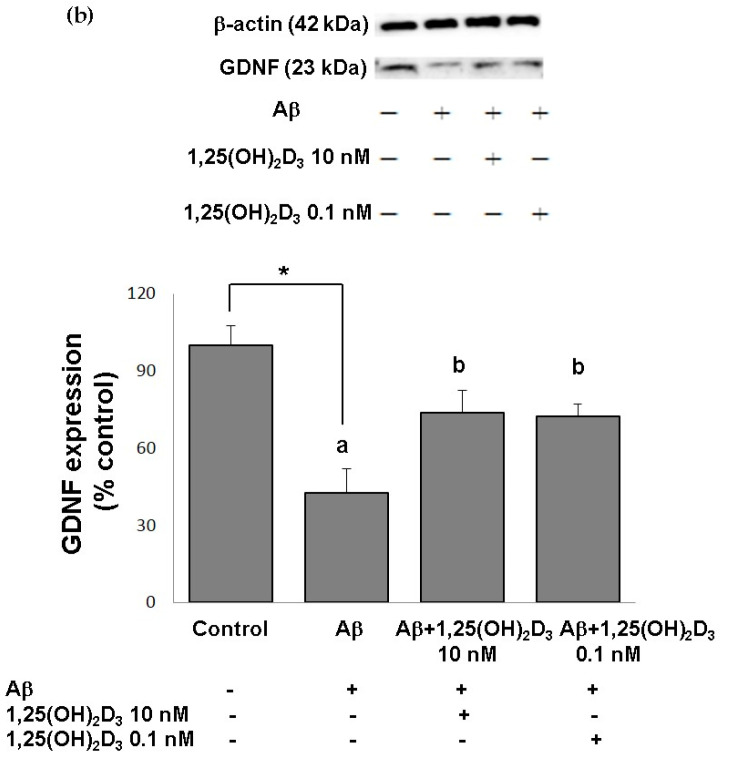
Effects of 1,25(OH)_2_D_3_ on Aβ-induced changes in vitamin D receptor (VDR) (**a**) and glial cell line-derived neurotrophic factor (GDNF) (**b**) protein expressions. SH-SY5Y cells were incubated with 1 μM Aβ(25-35) prior to the addition of 0.1 and 10 nM 1,25(OH)_2_D_3_ for 24 h.* Significantly differs from the control group (statistical analysis was performed using Student’s t test). Bars of Aβ, Aβ + 0.1 nM 1,25(OH)_2_D_3_, and Aβ + 10 nM 1,25(OH)_2_D_3_ with different letters significantly differ from each other (*p* < 0.05) (statistical analysis was performed using one-way analysis of variance (ANOVA) with Duncan’s post-hoc analysis). All data are expressed as mean ± SD of three experiments, and each experiment included triplicate repeats.

In this study, microscopic observation revealed overt abnormality of SH-SY5Y cell morphology after Aβ(25-35) stimulation, in which round-shaped cells without the extension of neurites were observed (shown by red arrows in Figure 1a), implying the deteriorating effect induced by Aβ(25-35) on neurite extension. However, as shown in Figure 1a, an angular shape and longer neurites of SH-SY5Y cells after 1,25(OH)_2_D_3_ treatment for 24 h were seen, suggesting that neurite extension was promoted by 1,25(OH)_2_D_3_. Moreover, the results from the MTT assay showed that Aβ(25-35) exposure significantly reduced cell viability when compared to the control group, whilst post-treatment with 1,25(OH)_2_D_3_ blocked this effect (Figure 1b). Further, to understand the toxic effects of Aβ(25-35) on protein expressions of VDR and GDNF, Western blotting analysis was employed. It was observed that Aβ(25-35) significantly decreased VDR (Figure 2a) and GDNF (Figure 2b) protein expressions compared to the control group (*p* < 0.05). Different dosages of 1,25(OH)_2_D_3_, 0.1 and 10 nM, were added after the Aβ(25-35) treatment, and both dosages significantly increased VDR and GDNF protein expressions compared to the Aβ(25-35) group (*p* < 0.05) (Figure 2). These results suggest that Aβ(25-35) was cytotoxic to the SH-SY5Y cells, leading to downregulations of VDR and GDNF, but these effects were able to be attenuated by 1,25(OH)_2_D_3_. Next, to verify the role of GDNF in mediating the neuroprotection of 1,25(OH)_2_D_3_ against Aβ(25-35) cytotoxicity, cells were then pretreated with Aβ(25-35) for 24 h prior to the addition of 1,25(OH)_2_D_3_ with or without the GDNF inhibitor, heparinase III, for 24 h. It was found that the presence of heparinase III significantly suppressed the cell viability induced by 1,25(OH)_2_D_3_ (Figure 1b). Altogether, these results support our hypothesis that the action of GDNF might be required for 1,25(OH)_2_D_3_-induced attenuation of cell survival evoked by Aβ(25-35).

### 2.2. Effects of 1,25(OH)_2_D_3_ on Activating Caspase-3 and Cell Apoptosis after Aβ(25-35) Treatment

**Figure 3:**
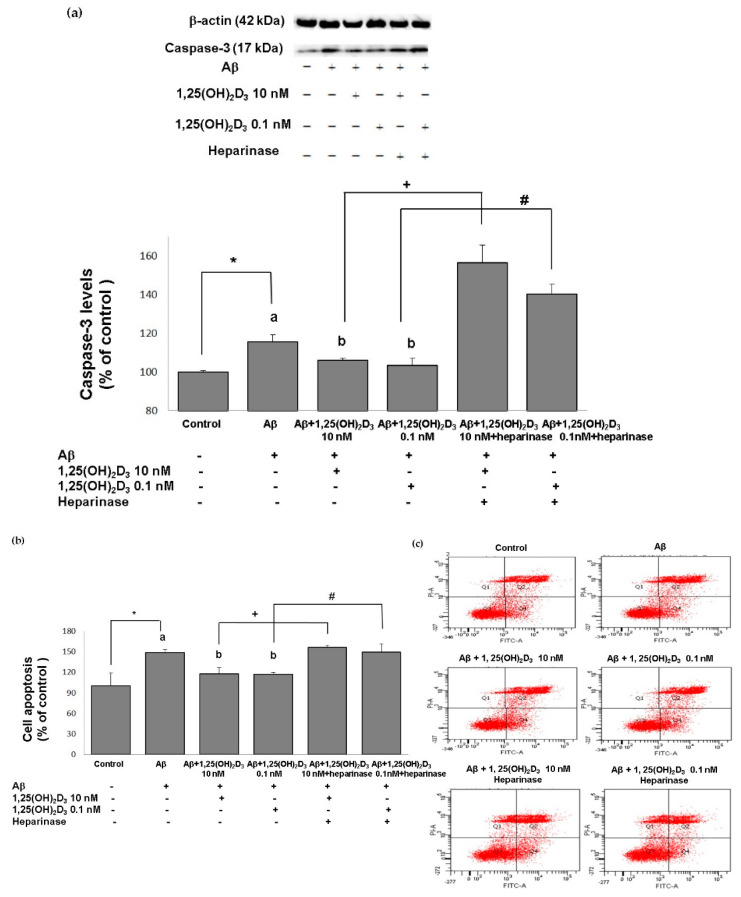
Effects of 1,25(OH)_2_D_3_ on Aβ-induced changes in cell apoptosis. (**a**) Western blot analysis of caspase-3 protein expression in SH-SY5Y cells. (**b**) Percentages of apoptotic cells in each group quantified from (**c**). (**c**) Representative profiles of cell apoptosis detected by flow cytometry with Annexin V/propidium iodide double-staining. SH-SY5Y cells were incubated with 1 μM Aβ(25-35) prior to the addition of 0.1 and 10 nM 1,25(OH)_2_D_3_ with or without heparinase III for 24 h. Data are presented as the mean ± SD of three experiments and each experiment included triplicate repeats. *^,+,#^ Significantly differs between the two groups (statistical analysis was performed using Student’s t test). Bars of Aβ, Aβ + 0.1 nM 1,25(OH)_2_D_3_, and Aβ + 10 nM 1,25(OH)_2_D_3_ with different letters significantly differ (*p* < 0.05) (statistical analysis was performed using one-way analysis of variance (ANOVA) with Duncan’s post-hoc analysis).

The neuroprotective role of 1,25(OH)_2_D_3_ was also validated by the apoptosis-related approaches, and similar results were revealed. The group treated with Aβ(25-35) exhibited significantly increased expression of activated caspase-3, a marker of cell death (Figure 3a), compared to the control group (*p* < 0.05), along with significant promotion of cell apoptosis (*p* < 0.05, Figure 3b,c). When 1,25(OH)_2_D_3_ was added after Aβ(25-35) treatment, it significantly decreased activated caspase-3 expression and cell apoptosis, compared to those of the Aβ(25-35) group (*p* < 0.05, Figure 3). These findings demonstrate that the Aβ(25-35) exposure resulted in apoptotic cell death, and this effect was attenuated by the 1,25(OH)_2_D_3_ treatment_._ In addition, Aβ(25-35)-induced apoptotic cell death and caspase-3 activation was unaffected by the 1,25(OH)_2_D_3_ treatment in the presence of heparinase III (Figure 3). Altogether, these observations support that the neuroprotective effects of 1,25(OH)_2_D_3_ on Aβ(25-35)-induced apoptosis might be elicited through the action of GDNF.

### 2.3. Effects of 1,25(OH)_2_D_3_ on Intracellular ROS after Aβ(25-35) Treatment

**Figure 4:**
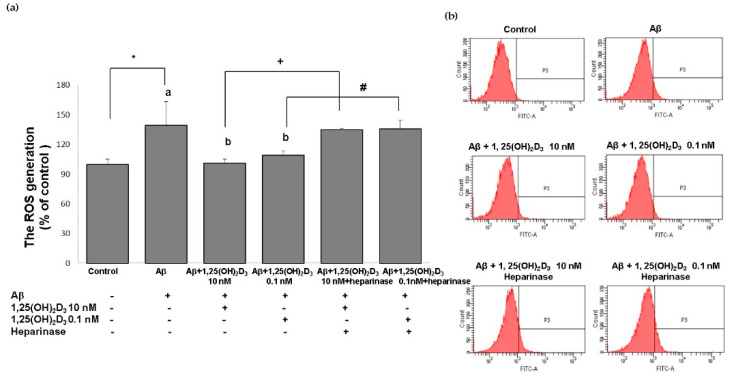
Effects of 1,25(OH)_2_D_3_ on Aβ-induced intracellular reactive oxygen species (ROS) production. (**a**) Quantitative results of ROS levels in each group according to (b). (**b**) Representative profiles of the intracellular ROS levels detected by flow cytometry using the 2’,7’-dichlorofluoroescin diacetate (DCFH-DA) assay. SH-SY5Y cells were incubated with 1 μM Aβ(25-35) prior to the addition of 0.1 and 10 nM 1,25(OH)_2_D_3_ with or without heparinase III for 24 h. Data are presented as the mean ± SD of three experiments, and each experiment included triplicate repeats. *^,+,#^ Significantly differs between the two groups (statistical analysis was performed using Student’s t test). Bars of Aβ, Aβ + 0.1 nM 1,25(OH)_2_D_3_, and Aβ + 10 nM 1,25(OH)_2_D_3_ with different letters significantly differ (*p* < 0.05) (statistical analysis was performed using one-way analysis of variance (ANOVA) with Duncan’s post-hoc analysis).

One promising mechanism underlying Aβ-induced apoptotic cell death is attributed to excessive production of ROS [33]. In order to better understand the neuroprotective mechanisms of 1,25(OH)_2_D_3_, in this study, the intracellular ROS levels were determined in SH-SY5Y cells using the DCF-DA assay. Figure 4 shows that the group treated with Aβ(25-35) exhibited significantly increased intracellular ROS production compared to the control group (*p* < 0.05). When 1,25(OH)_2_D_3_ at 0.1 and 10 nM was added after Aβ treatment, it caused a significant decrease in intracellular ROS compared to the Aβ group (*p* < 0.05). Heparinase III counteracted the effect of 1,25(OH)_2_D_3_ on intracellular ROS generation (*p* < 0.05, Figure 4). From these results, 1,25(OH)_2_D_3_ could significantly scavenge intracellular ROS triggered by Aβ(25-35), suggesting an antioxidant potential of 1,25(OH)_2_D_3_. Furthermore, these results support our central hypothesis that the role of GDNF was closely associated with the antioxidative effect of 1,25(OH)_2_D_3_ against Aβ(25-35)-induced intracellular ROS generation.

### 2.4. Effects of 1,25(OH)_2_D_3_ on the p-Tau/Tau Ratio after Aβ(25-35) Treatment

**Figure 5:**
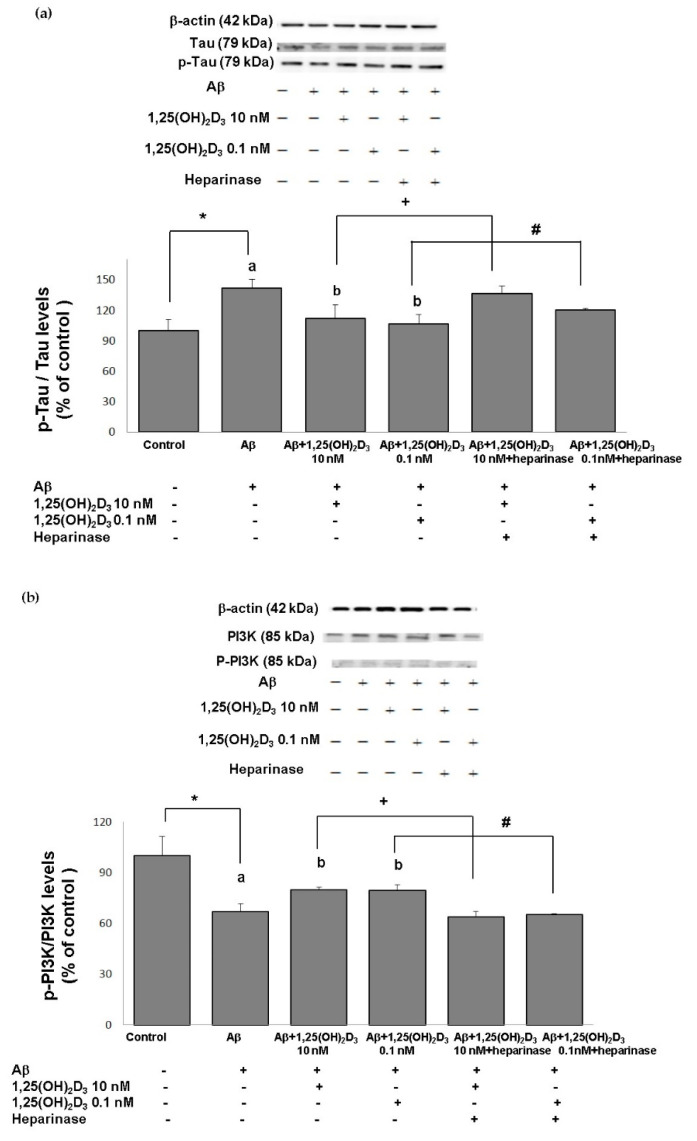

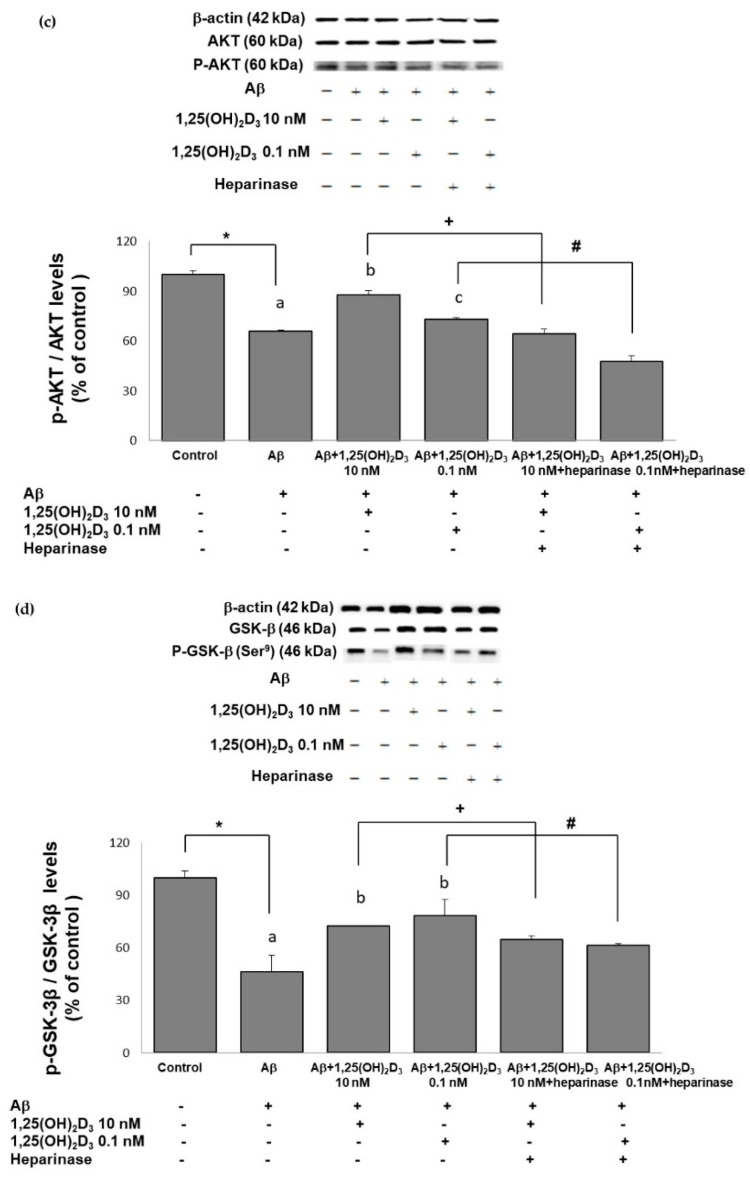
Effects of 1,25(OH)_2_D_3_ on Aβ-induced changes in the phosphorylated (p)-tau/tau ratio (**a**), phosphorylated (p)-phosphatidylinositol 3K (PI3K)/PI3K ratio (**b**), the phosphorylated (p)-Akt/Akt ratio (**c**), and the phosphorylated (p)-glycogen synthase kinase (GSK)-3β (Ser^9^)/GSK-3β ratio (**d**) of protein expressions. SH-SY5Y cells were incubated with 1 μM Aβ(25-35) prior to the addition of 0.1 and 10 nM 1,25(OH)_2_D_3_ with or without heparinase III for 24 h. Data are presented as the mean ± SD of three experiments, and each experiment included triplicate repeats. *^,+,#^ Significantly differs between the two groups (statistical analysis was performed using Student’s t test). Bars of Aβ, Aβ + 0.1 nM 1,25(OH)_2_D_3_, and Aβ + 10 nM 1,25(OH)_2_D_3_ with different letters significantly differ (*p* < 0.05) (statistical analysis was performed using one-way analysis of variance (ANOVA) with Duncan’s post-hoc analysis).

Excess generation of ROS has been shown to play a crucial role in the mechanisms associated with Aβ-induced neurotoxicity as well as tau pathology [34,35]. Herein, we first examine the level of tau phosphorylation in cells exposed to Aβ(25-35) prior to 1,25(OH)_2_D_3_ treatment. The group treated with Aβ exhibited a significant increase in the p-tau/tau ratio compared to the control group (Figure 5a). When 1,25(OH)_2_D_3_ at 0.1 and 10 nM was added after the Aβ(25-35) treatment, it caused a significant decrease in the p-tau/tau ratio compared to the Aβ group (*p* < 0.05, Figure 5a). The presence of heparinase III significantly increased the p-tau/tau ratio (*p* < 0.05, Figure 5a). These results show that 1,25(OH)_2_D_3_ was able to inhibit Aβ(25-35)-stimulated tau phosphorylation, and this action was linked to GDNF action. 

### 2.5. Effects of 1,25(OH)_2_D_3_ on the p-PI3K/PI3K, p-Akt/Akt, and p-GSK-3β (Ser^9^)/GSK-3β Ratios after Aβ(25-35) Treatment

One of the putative cellular mechanisms by which Aβ(25-35) induces ROS and apoptotic cell death involves the aforementioned dysregulation of the PI3K/Akt/GSK-3β signaling pathway. Therefore, we next examined the neuroprotective effects of 1,25(OH)_2_D_3_ on several proteins of this pathway after Aβ(25-35) exposure by the use of Western blotting analysis. The group treated with Aβ(25-35) exhibited significant decreases in the p-PI3K/PI3K (Figure 5b), p-Akt/Akt (Figure 5c), and p-GSK-3β (Ser^9^)/GSK-3β (Figure 5d) ratios compared to the control group (*p* < 0.05). Treatment with 1,25(OH)_2_D_3_ at 0.1 and 10 nM after Aβ(25-35) treatment significantly increased the phosphorylation of these proteins compared to the Aβ(25-35) group (*p* < 0.05, Figure 5). However, with the presence of heparinase III, a GDNF-signaling inhibitor, the effect of 1,25(OH)_2_D_3_ on phosphorylation was reduced (*p* < 0.05, Figure 5). These results revealed that 1,25(OH)_2_D_3_ was able to stimulate Aβ(25-35)-inhibited phosphorylation of PI3K, Akt, and GSK-3β, and such stimulation appeared to be related to GDNF action.

## 3. Discussion

It is recognized that aberrant Aβ exhibits neurotoxicity that contributes to neuronal death, and this event is thought to be the primary factor that initiates the pathogenesis of AD [36,37]. Several neurotoxic effects of Aβ shown in the present study are consistent with previous studies [38,39]. For instance, we observed changes in cell morphology and tau phosphorylation, and increase in the number of apoptotic cells in parallel with the excess generation of ROS after Aβ treatment. These findings support that Aβ-associated oxidative stress was involved in the observed neuronal damage, and thus played an important role in Aβ neurotoxicity [34]. Furthermore, the morphology of neuronal cells is stabilized by the tau protein [40]. Once the tau protein is hyperphosphorylated, as observed after Aβ treatment in this study, it failed to maintain the cell structure [41] and caused cell apoptosis [2,42,43]. It is worth mentioning that neurons are capable of protecting against oxidative damage through secreting neurotrophic factors; as neurotrophic factors decrease, neurons are unable to eliminate the accumulated ROS [15,44]. In this study, we observed that both ROS production and cellular apoptosis increased as GDNF expression decreased after Aβ treatment. Hence, we speculate that Aβ might exert its toxic effects by inhibiting the action of GDNF and augmenting oxidative stress and apoptosis. In this regard, given the strong implication of excessive production of ROS and the reduction in GDNF levels in the mechanisms of Aβ neurotoxicity, it is plausible that antioxidants could be effective in the treatment of Aβ-related pathological processes [45].

In recent years, 1,25(OH)_2_D_3_ has received great attention due to its therapeutic potential as a potent antioxidant and neuroprotectant [24,46]. In the brain, 1,25(OH)_2_D_3_ regulates the neurotrophic factors via VDR, thereby controlling neuronal survival, development, and function [47]. There is evidence that protein and gene expressions of GDNF can be elevated by the binding of 1,25(OH)_2_D_3_ to the VDR [48,49]. As 1,25(OH)_2_D_3_ binds to the VDR, the protein and gene expressions of the VDR also increase [50,51]. In contrast, it was found that Aβ suppresses the protein and gene expressions of the VDR [52]. In the present study, we first confirmed that VDR and GDNF expressions were both suppressed by Aβ treatment in our model. This suppression of VDR and GDNF expression was reversed after the addition of 1,25(OH)_2_D_3_, indicating that the upregulation of GDNF may be a consequence of the formation of the 1,25(OH)_2_D_3_/VDR complex. These data suggest that VDR activity may be linked to GDNF production [53]. Taken together, we hypothesized that for 1,25(OH)_2_D_3_ to elicit its anti-Aβ cytotoxicity, GDNF-signaling may be required as a cooperating event. Our hypothesis is supported by a previous study reporting that a GDNF mechanism potentially participates in anti-neurotoxicity of 1,25(OH)_2_D_3_, regardless of the type of toxic substances administered [54]. Our observations described below corroborate such statements. Suppression of ROS production and apoptotic cell death after the administration of 1,25(OH)_2_D_3_ supports the theory that 1,25(OH)_2_D_3_ may act as an antioxidant as well as a neuroprotectant to ameliorate Aβ-induced oxidative damage [22]. To determine whether this protection involves the upregulation of GDNF in response to 1,25(OH)_2_D_3_, we utilized heparinase III to block GDNF signaling and found that the generation of ROS was indeed not affected. A recent study has established that GDNF-signaling in dopaminergic neurons is regulated by 1,25(OH)_2_D_3_ [55], which supports our discovery of an interplay between 1,25(OH)_2_D_3_-VDR and GDNF signaling. We therefore postulate that the ability of 1,25(OH)_2_D_3_ to decrease ROS produced by Aβ may occur, at least in part, through direct interactions of 1,25(OH)_2_D_3_ with GDNF at the cellular levels.

In an attempt to further understand the interaction between 1,25(OH)_2_D_3_-VDR and GDNF-signaling against Aβ neurotoxicity, the PI3K/AKT/GSK-3β pathway was examined due to its involvement in the promotion of cell survival and the pathogenesis of AD [56,57]. The GDNF-stimulated PI3K/Akt pathway regulates phosphorylation of GSK-3β (Ser^9^) and cell survival [58,59,60,61]. It was indicated that Aβ also decreases GDNF secretion and increases activation of GSK-3β, promoting tau protein hyperphosphorylation and neuronal apoptosis in the brain [62,63,64,65,66,67]. Inactivation of Akt in the brain causes the amyloid protein precursor (APP) to accumulate [64]. In the present study, we observed that Aβ treatment downregulated the activated form of PI3K/Akt and that 1,25(OH)_2_D_3_ reversed this dysregulation. Akt is the main regulator of GSK-3β [9]. Activation of GSK-3β causes greater Aβ accumulation and promotion of cell apoptosis through caspase-3 activation [62,68]. Activation of GSK-3β decreases as Akt is phosphorylated (i.e., activated), which results in greater cell survival [62]. Importantly, the major cause of the decrease in activation of Akt is downregulation of neurotrophic factors [62]. In the present study, we found that Aβ treatment enhanced the activated forms of GSK-3β and caspase-3, and tau protein hyperphosphorylation, but 1,25(OH)_2_D_3_ administration normalized these hyperactivations caused by Aβ. Altogether, in the present study, 1,25(OH)_2_D_3_ potentiated PI3K/Akt activation and subsequently led to the inactivation of downstream GSK-3β upon Aβ challenge. These findings demonstrate a putative neuroprotective role of 1,25(OH)_2_D_3_ against Aβ neurotoxicity by acting on the PI3K/AKT/GSK-3β pathway. In addition, blockage of the GDNF upregulation by heparinase III was likely to prevent the aforementioned beneficial effects of 1,25(OH)_2_D_3_. Our data suggest that the protective PI3K/AKT/GSK-3β pathway involving GSK-3β inactivation may be partially mediated through GDNF [65]. Therefore, we propose that GDNF signaling might be an important driving mechanism underlying the 1,25(OH)_2_D_3_-mediated modulating effects on Aβ-induced neurotoxicity.

As mentioned previously, PI3K/AKT pathway activation mediated by GDNF contributes to neuronal survival, making cells resistant to apoptosis [58]. In this study, PI3K/Akt downregulation may have resulted in activation of GSK-3β and caspase-3, thus inducing cell apoptosis. Aβ accumulation to cause toxicity may exacerbate neuronal damage, leading to cell apoptosis [69]. In our study, treatment with 1,25(OH)_2_D_3_ appeared to attenuate Aβ-induced apoptosis, supporting that 1,25(OH)_2_D_3_ may be anti-apoptotic. In addition, combinational treatment with1,25(OH)_2_D_3_ and the GDNF inhibitor showed inhibition of the counteracting of apoptosis in the presence of Aβ. As a possible consequence to these findings, upregulation of GDNF seemed to be a key mechanism through which 1,25(OH)_2_D_3_ neutralized Aβ-induced excessive ROS production and apoptotic death in SH-SY5Y cells. Collectively, 1,25(OH)_2_D_3_ might elicit its neuroprotection via actions of GDNF-signaling, with the signals subsequently and indirectly leading to the resistance of SH-SY5Y cells to Aβ neurotoxicity.

## 4. Materials and Methods 

### 4.1. Aβ(25-35) and 1,25(OH)_2_D_3_ Preparations

A short fragment of full length Aβ(1-42), Aβ(25-35) (A4559, Sigma, St. Louis, MO, USA), was employed in the present study due to the fact that it has been demonstrated to exist in AD brains [70], it exhibits the same neurotoxicity as Aβ(1-42), and has exhibited rapidly developed toxic effects in in vitro studies [10]. In brief, Aβ(25-35) was dissolved in sterile distilled water at a concentration of 1 mM as a stock solution before being diluted to desired concentrations. It was then incubated in capped vials at 37 °C for 5 days to form aggregates and develop full toxicity. It was stored frozen at −20 °C until use [71,72]. Next, the stock solution of Aβ(25-35) was diluted and added to cultures in a final concentration of 1 μM for 24 h prior to the treatment of 1,25(OH)_2_D_3_. The dose of Aβ(25-35) at 1 μM was chosen based on the literature [73] and the results of our preliminary experiments, in which cell viability, ROS, and apoptosis assays were conducted to test maximal toxic effects (data not shown). 

For the preparation of 1,25(OH)_2_D_3_, the biological concentration of vitamin D in the peripheral circulation in healthy people is around 20 ng/mL (50 nM) [74] and 10 nM was indicated to induce GDNF expression [48,49]. Considering the conversion rate from 25-hydroxyvitamin D_3_ to 1,25(OH)_2_D_3_ and much lower concentrations in the brain, we treated cells with 0.1 and 10 nM of 1,25(OH)_2_D_3_. For this, 1,25(OH)_2_D_3_ (D1530, Sigma, St. Louis, MO, USA) was dissolved in 99.5% ethanol to a concentration of 10 mM as a stock solution before being diluted in 99.5% ethanol to desired concentrations.

### 4.2. Cell Culture Preparation

The SH-SY5Y human neuroblastoma cell line is a well-established cell model for studying neurodegenerative diseases [75]. The cell line was purchased from American Type Culture Collection (ATCC, Manassas, VA, USA). Cells were cultured in Dulbecco’s modified Eagle medium (DMEM) mixed with F12 (Gibco, Paisley, UK), 10% fetal bovine serum, and sodium bicarbonate at 37 °C in a 5% CO_2_ incubator. The medium was replaced every 2~3 days. Each aliquot (vial) of cells was grown for no more than 10 passages. Experiments were performed at 80% cell confluence. Then, cells were incubated with 1 μM of Aβ(25-35) for 24 h, followed by washing and incubation with two different concentrations of 1,25(OH)_2_D_3_ (0.1 or 10nM) for 24 h. Heparinase III (H8891, Sigma, St. Louis, MO, USA), an inhibitor of the GDNF-signaling, was used with 1,25(OH)_2_D_3_ treatment in some of the experiments to elucidate the role of GDNF on 1,25(OH)_2_D_3_-stimulated effects.

### 4.3. Cell Morphology

Cell morphology was observed under a microscope (Nikon, Tokyo, Japan) at 40× and 400× magnifications, and photos were processed with SPOT 4.7 Advanced software (SPOT Imaging Solutions, Sterling Heights, MI, USA).

### 4.4. Cell Viability Analysis

A 3-[4,5-cimethylthiazol-2-yl]-2,5-diphenyl tetrazolium bromide (MTT) assay was performed to determine the viability of SH-SY5Y cells that were pre-treated with Aβ(25-35) for 24 h and then further treated with two different concentrations of 1,25(OH)_2_D_3_ for 24 h. Briefly, MTT was added to each well of a 24-well plate and incubated at 37 °C for 1 h. Purple-colored precipitates of the living cell metabolite, formazan, were then dissolved in 500 µL of dimethyl sulfoxide (DMSO) and were analyzed in a 96-well plate. The color absorbance was recorded at 590 nm. Cell viability was calculated by the absorbance ratio of the treated group over the control.

### 4.5. Intracellular ROS Analysis

The production of intracellular ROS was determined by the 2’,7’-dichlorofluoroescin diacetate (DCFH-DA) probe, which is converted to the fluorescent dichlorofluorescein (DCF) in the presence of peroxides. Cells were seeded in 6-well dishes at 5 × 10^5^ cells per well before the allotted experimental treatments were performed. After being treated, cells were trypsinized and washed with phosphate-buffered saline (PBS) once by centrifugation at 200× *g* for 3 min at 25 °C. After removing the supernatant, DCFH-DA dissolved in PBS was added to each sample. Samples were then incubated in the complete absence of light for 60 min. Each sample was moved to a Falcon tube prior to analysis by flow cytometry (Flowcytometer-3, FACSCantoII, BD Biosciences, Franklin Lake, NJ, USA).

### 4.6. Protein Extraction and Quantification

After being treated, cells were harvested, washed three times with PBS, and lysed using cold RIPA buffer supplemented with a protease inhibitor and an EDTA solution at a ratio of 100:1:1, respectively, then centrifuged at 13,000× *g* at 4 °C for 30 min. The supernatant was collected, and the protein concentration was estimated with a BCA Protein Assay Kit (Milpitas, CA, USA) using bovine serum albumin as the standard.

### 4.7. Western Blot Analysis

A Western blot analysis was performed to examine expression levels of certain proteins. Equal quantities (30 µg) of proteins were separated by 10% sodium dodecyl sulfate (SDS) polyacrylamide gel electrophoresis and then transferred onto nitrocellulose membranes. After being transferred, membranes were blocked with Tris-buffered saline (TBS) containing 0.1% Tween-20 (TBST) and 5% non-fat milk for 1 h. Membranes were subsequently incubated with specific primary antibodies: β-actin (A3854, Sigma, St. Louis, MO, USA), GDNF (MAB212, R&D System, Minneapolis, MN, USA), vitamin D receptor (VDR; ab8756, Abcam, Cambridge, MA, USA), phosphorylated (p)-phosphatidylinositol 3K (PI3K; 4228, Cell Signaling Technology, Danvers, MA, USA), PI3K (4292, Cell Signaling Technology), p-Akt (9271, Cell Signaling Technology), Akt (9272, Cell Signaling Technology), p-glycogen synthase kinase (GSK)-3β (Ser^9^) (9336, Cell Signaling Technology), GSK-3β (9315, Cell Signaling Technology), activated caspase-3 (ab13847, Abcam), p-tau (ab109390, Abcam), and tau (ab22261, Abcam) overnight at 4 °C. After washing three times with TBST for 30 min, the membranes were incubated with an anti-mouse (A9024, Sigma) or an anti-rabbit (R5506, Sigma) immunoglobulin G (IgG) secondary antibody for 1 h, and then washed with TBST three times for 30 min. Immunoreactive proteins were detected and quantified using an enhanced chemiluminescence (ECL; Bionovas, Toronto, Canada) Western blot detection system and Image-Pro Plus Software (Cybernetics, Rockville, MD, USA), respectively.

### 4.8. Apoptotic Cell Analysis

Apoptosis cell analyses were performed using flow cytometry by double-staining with propidium iodide (PI) and annexin-V dye. Cells were seeded in 6-well dishes at 5 × 10^5^ cells per well before the allotted experimental treatments were performed. After being treated, cells were trypsinized and washed with PBS at least twice by centrifugation at 200× *g* for 3 min at 4 °C. The supernatant was removed, and the pellet was re-suspended in 1 mL of cold PBS and centrifuged for 3 min at 200× *g* and 4 °C. After removing the supernatant, 100 µL of binding buffer, 2 µL of PI dye, and 2 µL of annexin-V dye were added to each sample. Samples were then incubated at room temperature in the complete absence of light for 15 min. Each sample was resuspended in 600 µL of cold PBS and moved to a Falcon tube prior to analysis by flow cytometry (Flowcytometer-3, FACSCantoII, BD Biosciences, San Jose, CA, USA).

### 4.9. Statistical Analysis

Statistical comparisons were performed with a *t*-test (control vs. Aβ; Aβ+1,25(OH)_2_D_3_ vs. Aβ+1,25(OH)_2_D_3_+heparinase) and one-way analysis of variance (ANOVA) with Duncan’s post-hoc analysis (Aβ vs. different concentrations of 1,25(OH)_2_D_3_). The level of significance was set at *p* < 0.05. Data are presented as the mean value and standard deviation (SD).

## 5. Conclusions

In conclusion, this study demonstrates the neuroprotective effects of 1,25(OH)_2_D_3_ against Aβ neurotoxicity and concomitant changes in phosphorylated tau protein in SH-SY5Y cells. The underlying mechanisms of the action of 1,25(OH)_2_D_3_ were attributed to its ability to counteract excessive production of ROS and apoptotic cell death. However, the optimal responses of 1,25(OH)_2_D_3_ to Aβ neurotoxicity in SH-SY5Y cells required an interplay with GDNF-signaling by targeting inactivation of the PI3K/Akt/GSK-3β pathway at the cellular level. However, more studies are warranted to explore the additional pathways through which GDNF works to mediate neuroprotection of 1,25(OH)_2_D_3_ and to better understand the interactions with the PI3K/Akt/GSK-3β signaling in relation to Aβ neurotoxicity. Nonetheless, we have demonstrated the pivotal role of GDNF in 1,25(OH)_2_D_3_-elicited neuroprotection following an Aβ challenge in SH-SY5Y cells, which could add value as the basis of 1,25(OH)_2_D_3_ treatment for limiting Aβ-related pathology.

## Abbreviations

Aβ	Amyloid beta
AD	Alzheimer’s disease
1,25(OH)_2_D_3_	1α,25-dihydroxyvitamin D3
VDR	Vitamin D receptor
ROS	Reactive oxygen species
GDNF	Glial cell line-derived neurotrophic factor
PI3K	Phosphoinositide 3-kinase
Akt	Protein kinase B
GSK-3β	Glycogen synthase kinase-3β
APP	Amyloid protein precursor
DCFH-DA	2′,7′-dichlorodihydrofluorescein diacetate
DMEM	Dulbecco’s modified Eagle medium
DCF	Dichlorofluorescein
MTT	3-[4,5-cimethylthiazol-2-yl]-2,5-diphenyl tetrazolium bromide
PBS	Phosphate-buffered saline
DMSO	Dimethyl sulfoxide
SDS	Sodium dodecyl sulfate
TBS	Tris-buffered saline
TBST	Tris-buffered saline containing 0.1% Tween-20
ECL	Enhanced chemiluminescence
IgG	Immunoglobulin G
PI	Propidium iodide
ANOVA	Analysis of variance
SD	Standard deviation
